# Supplementary material for: The Effects of Maternal Iron and Folate Supplementation on Pregnancy and Infant Outcomes in Africa: A Systematic Review
Source: Int J Environ Res Public Health. 2024 Jun 29;21(7):856. doi: 10.3390/ijerph21070856 (PMC11276896; doi:10.3390/ijerph21070856)
Supplement: Supplementary file 1 [file ijerph-21-00856-s001.zip › ijerph-3011166-supplementary.pdf]

The effects of maternal oral iron and folate supplementation on pregnancy and infant outcomes in Africa: A systematic review.

Yibeltal Bekele (YB)[1,4], Claire Gallagher (CG)[2], Don Vicendese (DV)[2,3], Melissa Buultjens (MB)[1], Mehak Batra (MB) [1*], Bircan Erbas (BE)[1*]

*Equal senior author.

[1]School of Psychology and Public Health, La Trobe University, Melbourne, VIC 3086, Australia.

[2]School of Population and Global Health, The University of Melbourne, VIC 3010, Australia.

[3]School of Computing, Engineering and Mathematical Sciences, La Trobe University, Melbourne, VIC 3086, Australia.

[4] School of Public Health, Bahir Dar University, Bahir Dar 79, Ethiopia.

YB: Y.Bekele@latrobe.edu.au

CG: claire-gallagher@live.com

DV: don.vicendese@unimelb.edu.au

MB: M.Buultjens@latrobe.edu.au

MB: M.batra@latrobe.edu.auu

BE: B.Erbas@latrobe.edu.au

Corresponding author:

Yibeltal Bekele, School of Psychology and Public Health, La Trobe University, Bundoora, Victoria, Australia.

Email: Y.Bekele@latrobe.edu.au

ORCID Number: orcid.org/0000-0003-1272-7824

Table S1: Prisma-P 2020 Checklist

Section and Topic	Item #	Checklist item	Page #
**TITLE**			
Title	1	Identify the report as a systematic review.	1
**ABSTRACT**			
Abstract	2	See the PRISMA 2020 for Abstracts checklist.	2
**INTRODUCTION**			
Rationale	3	Describe the rationale for the review in the context of existing knowledge.	3 to 5
Objectives	4	Provide an explicit statement of the objective(s) or question(s) the review addresses.	5
**METHODS**			
Eligibility criteria	5	Specify the inclusion and exclusion criteria for the review and how studies were grouped for the syntheses.	5&6
Information sources	6	Specify all databases, registers, websites, organisations, reference lists and other sources searched or consulted to identify studies. Specify the date when each source was last searched or consulted.	6
Search strategy	7	Present the full search strategies for all databases, registers and websites, including any filters and limits used.	6
Selection process	8	Specify the methods used to decide whether a study met the inclusion criteria of the review, including how many reviewers screened each record and each report retrieved, whether they worked independently, and if applicable, details of automation tools used in the process.	6 & 7
Data collection process	9	Specify the methods used to collect data from reports, including how many reviewers collected data from each report, whether they worked independently, any processes for obtaining or confirming data from study investigators, and if applicable, details of automation tools used in the process.	7
Data items	10a	List and define all outcomes for which data were sought. Specify whether all results that were compatible with each outcome domain in each study were sought (e.g. for all measures, time points, analyses), and if not, the methods used to decide which results to collect.	6 Table 1
	10b	List and define all other variables for which data were sought (e.g. participant and intervention characteristics, funding sources). Describe any assumptions made about any missing or unclear information.	6 & Table 1
Study risk of bias assessment	11	Specify the methods used to assess risk of bias in the included studies, including details of the tool(s) used, how many reviewers assessed each study and whether they worked independently, and if applicable, details of automation tools used in the process.	7
Effect measures	12	Specify for each outcome the effect measure(s) (e.g. risk ratio, mean difference) used in the synthesis or presentation of results.	7 & Table 2
Synthesis methods	13a	Describe the processes used to decide which studies were eligible for each synthesis (e.g. tabulating the study intervention characteristics and comparing against the planned groups for each synthesis (item #5)).	7

Table S1: Prisma-P 2020 Checklist

Section and Topic	Item #	Checklist item	Page #
	13b	Describe any methods required to prepare the data for presentation or synthesis, such as handling of missing summary statistics, or data conversions.	-
	13c	Describe any methods used to tabulate or visually display results of individual studies and syntheses.	-
	13d	Describe any methods used to synthesize results and provide a rationale for the choice(s). If meta-analysis was performed, describe the model(s), method(s) to identify the presence and extent of statistical heterogeneity, and software package(s) used.	-
	13e	Describe any methods used to explore possible causes of heterogeneity among study results (e.g. subgroup analysis, meta-regression).	-
	13f	Describe any sensitivity analyses conducted to assess robustness of the synthesized results.	-
Reporting bias assessment	14	Describe any methods used to assess risk of bias due to missing results in a synthesis (arising from reporting biases).	-
Certainty assessment	15	Describe any methods used to assess certainty (or confidence) in the body of evidence for an outcome.	-
**RESULTS**			
Study selection	16a	Describe the results of the search and selection process, from the number of records identified in the search to the number of studies included in the review, ideally using a flow diagram.	9 & Figure 1
	16b	Cite studies that might appear to meet the inclusion criteria, but which were excluded, and explain why they were excluded.	Supplementary material
Study characteristics	17	Cite each included study and present its characteristics.	Table 1
Risk of bias in studies	18	Present assessments of risk of bias for each included study.	Supplementary material
Results of individual studies	19	For all outcomes, present, for each study: (a) summary statistics for each group (where appropriate) and (b) an effect estimates and its precision (e.g. confidence/credible interval), ideally using structured tables or plots.	Table 2
Results of syntheses	20a	For each synthesis, briefly summarise the characteristics and risk of bias among contributing studies.	9 to 17
	20b	Present results of all statistical syntheses conducted. If meta-analysis was done, present for each the summary estimate and its precision (e.g. confidence/credible interval) and measures of statistical heterogeneity. If comparing groups, describe the direction of the effect.	-
	20c	Present results of all investigations of possible causes of heterogeneity among study results.	-
	20d	Present results of all sensitivity analyses conducted to assess the robustness of the synthesized results.	-
Reporting biases	21	Present assessments of risk of bias due to missing results (arising from reporting biases) for each synthesis assessed.	-
Certainty of evidence	22	Present assessments of certainty (or confidence) in the body of evidence for each outcome assessed.	-
**DISCUSSION**			
Discussion	23a	Provide a general interpretation of the results in the context of other evidence.	17 to 19
	23b	Discuss any limitations of the evidence included in the review.	17 to 19

Table S1: Prisma-P 2020 Checklist

Section and Topic	Item #	Checklist item	Page #
	23c	Discuss any limitations of the review processes used.	19
	23d	Discuss implications of the results for practice, policy, and future research.	17 to 19
**OTHER INFORMATION**			
Registration and protocol	24a	Provide registration information for the review, including register name and registration number, or state that the review was not registered.	5
	24b	Indicate where the review protocol can be accessed, or state that a protocol was not prepared.	5
	24c	Describe and explain any amendments to information provided at registration or in the protocol.	-
Support	25	Describe sources of financial or non-financial support for the review, and the role of the funders or sponsors in the review.	-
Competing interests	26	Declare any competing interests of review authors.	20
Availability of data, code, and other materials	27	Report which of the following are publicly available and where they can be found: template data collection forms; data extracted from included studies; data used for all analyses; analytic code; any other materials used in the review.	-

Table S2: Preliminary search strategy from MEDLINE.

No.	Concept	Search terms
1	Pregnant women	pregnancy/ or exp gravidity/ or exp labor, obstetric/ or exp parity/ or exp parturition/ or exp placentation/ or exp pregnancy, multiple/ or exp pregnancy, twin/ or exp superfetation/ or exp pregnancy, unplanned/ or exp pregnancy, unwanted/ or Pregnant Women/or mothers/ or exp single parent/ or exp surrogate mothers/ or adolescent mothers/ or perinatal care/ or exp postnatal care/ or exp preconception care/ or exp prenatal care/ or (pregnan* or wom#n* or mother* or mom* or matern* or partus or lactation or labo#r or childbear* or child-bear* or gestation* or antenatal or ante-natal or "ante natal" or pre-natal or prenatal or peri-natal or perinatal or peri-partum or "peri partum" or "child birth" or child-birth or childbirth or "term birth")
2	Iron folate supplementation	Micronutrients supp/ or exp trace elements/ or exp iron/ or exp vitamins/ or exp folic acid/ or ("Iron folate supplementation" or "Iron and folate supplementation" or "iron supplementation" or "folic acid supplementation")
3	Adverse birth outcomes/neonatal /infant mortality	pregnancy complications/ or exp abortion, spontaneous/ or exp abortion, habitual/ or exp abortion, incomplete/ or exp abortion, missed/ or exp abortion, septic/ or exp abortion, threatened/ or exp fetal death/ or exp stillbirth/ or exp perinatal death/ or infant/ or exp infant, newborn/ or exp infant, low birth weight/ or exp infant, small for gestational age/ or exp infant, very low birth weight/ or exp infant, extremely low birth weight/ or exp infant, postmature/ or exp infant, premature/ or exp infant, extremely premature/ or infant mortality/ or ("pregnanc* complication*" or "low birth weight" or stillbirth or "still birth" or "low birth weight" or "preterm birth" or miscarriage or abortion or "prenatal mortality" or "perinatal mortality" or "very low small for weight" or "small for gestational age" or "newborn death" or "early neonatal mortality" or "late neonatal mortality" or "post neonatal mortality" or "neonatal mortality" or "post neonatal mortality" or "infant mortality").mp.
4	Africa	Africa/ or exp africa, northern/ or exp algeria/ or exp egypt/ or exp libya/ or exp morocco/ or exp tunisia/ or exp "africa south of the sahara"/ or exp africa, central/ or exp cameroon/ or exp central african republic/ or exp chad/ or exp congo/ or exp "democratic republic of the congo"/ or exp equatorial guinea/ or exp gabon/ or exp "sao tome and principe"/ or exp africa, eastern/ or exp burundi/ or exp comoros/ or exp djibouti/ or exp eritrea/ or exp ethiopia/ or exp kenya/ or exp madagascar/ or exp rwanda/ or exp seychelles/ or exp somalia/ or exp south sudan/ or exp sudan/ or exp tanzania/ or exp uganda/ or exp africa, southern/ or exp angola/ or exp botswana/ or exp eswatini/ or exp lesotho/ or exp malawi/ or exp mozambique/ or exp namibia/ or exp south africa/ or exp zambia/ or exp zimbabwe/ or exp africa, western/ or exp benin/ or exp burkina faso/ or exp cabo verde/ or exp cote d'ivoire/ or exp gambia/ or exp ghana/ or exp guinea/ or exp guinea-bissau/ or exp liberia/ or exp mali/ or exp mauritania/ or exp niger/ or exp nigeria/ or exp senegal/ or exp sierra leone/ or exp togo/ or ("Sub Sahara Africa" or Africa or "central Africa" or "western Africa" or "east Africa" or "southern Africa" or "Norther Africa" or "Burkina Faso" or Burundi or "Central Africa Republic" or Chad or "DR Congo" or Eritrea or Ethiopia or Gambia or Guinea or Guinea-bisseau or Liberia or Madagascar or Malawi or Mali or Mozambique or Niger or Rwanda or "Sierra Leone" or Somalia or "South Sudan" or Sudan or Togo or Uganda or Zambia or Algeria or Angola or Benin or Cameroon or "Cape Verde" or Comoros or Djibouti or Egypt or Eswatini or Ghana or "Ivory Coast" or Kenya or Lesotho or Mauritania or Morocco or Nigeria or "Republic of the Congo" or Senegal or Tanzania or Tunisia or Zimbabwe or Botswana or "Equatorial Guinea" or Gabon or Libya or Mauritius or Namibia or "South Africa")
**5**	Combination	1 AND 2 AND 3 AND 4

Table S3: Quality Assessment results of included studies.

Quality Assessment result of cross-sectional studies

	Selection				Comparability		Outcome			
Authors/Year	Representativeness of the sample	Sample size (is the sample size justifiable and	Non-respondents	Ascertainment of the exposure (risk factor)	The study controls for the most important factor (ANC, Residence, maternal age, maternal educational status)	The study control for any additional factor	Assessment of outcome	Statistical test	Total	Grade
Godha et al, 2022	*	*	*	**	*		*	*	8	low risk
Titaley, et al. 2010	*	*	*	**	*		*	*	8	Low risk
Ali et al. 2014	*	*	*	*	*		*	*	7	Low risk
Lolaso et al. 2021	*	*	*	**	*		**	*	9	Low risk

Quality assessment result of Case-control studies

	Selection				Comparability		Exposure				
Authors/Year	Is the Case Definition Adequate?	Representativeness of the Cases	Selection of Controls	Definition of Controls	Controls for the most important factor (ANC, Residence, maternal age, maternal educational [illegible]	The study control for any other or additional factors	exposures were measured using standard tools, medical records, or [illegible]	The same method of ascertainment for cases and controls	Non-Response Rate	Total	Grade
Goba et al. 2017	*	*	*	*	*	*		*	*	8	Low risk
Hailemichael et al. 2020		*		*	*	*		*		6	Medium risk

Risk of bias assessment result for Cohort study design

	Selection				Comparability		Exposure				
Authors/year	Representativeness of the exposed cohort	Selection of the Non-Exposed Cohort	Ascertainment of Exposure	Demonstration that outcome of interest was not present at the start of study(yes/no)	study controls for (ANC, Residence, maternal age, maternal educational status)	study controls for any additional factor	Assessment of Outcome	was wollow-up long enough for outcomes to occur	Adequacy of follow-up of cohorts (respondents)	Total	Grade
Ibrahim et al. 1994	*	*		*			*	*		5	Medium risk
Kone et al. 2018	*	*	*	*	*		*	*	*	8	Low risk
Zelka et al. 2023	*	*	*	*	*	*	*	*		7	Low risk

Chalumeau et al. 2002	*		*	*	*		*	*	*	7	Low risk
